# Li^+^ Influx and Binding, and Li^+^/Mg^2+^ Competition in Bovine Chromaffin Cell Suspensions as Studied by ^7^Li NMR and Fluorescence Spectroscopy

**DOI:** 10.1155/MBD.2000.357

**Published:** 2000

**Authors:** Carla P. Fonseca, Liliana P. Montezinho, Graça Baltazar, Brian Layden, Duarte M. Freitas, Carlos F. G. C. Geraldes, M. Margarida C. A. Castro

**Affiliations:** 1 Department of Biochemistry University of Coimbra P.O. Box 3126 Coimbra 3000 Portugal; 2 Center for Neuroscience University of Coimbra P.O. Box 3126 Coimbra 3000 Portugal; 3 Chemistry Department Loyola University of Chicago Chicago IL 60626 USA

## Abstract

Li^+^ influx by bovine chromaffin cells, obtained from bovine adrenal medulla, was studied in intact cell suspensions using  ^7^Li NMR spectroscopy with the shift reagent [Tm(HDOTP)]^4-^. The influx rate constants, k_i_, were determined in the absence and in the presence of two Na^+^ membrane transport inhibitors. The values obtained indicate that both voltage sensitive Na^+^ channels and (Na^+^/K^+^)-ATPase play an important role in Li^+^ uptake by these cells. ^7^Li NMR T_1_ and T_2_ relaxation times for intracellular Li^+^ in bovine chromaffin cells provided a T_1_/T_2_ ratio of 305, showing that Li^+^ is highly, immobilized due to strong binding to intracellular structures. Using fluorescence spectroscopy and the Mg^2+^ fluorescent probe, furaptra, the free intracellular Mg^2+^ concentration in the bovine chromaffin cells incubated with 15 mM LiCl was found to increase by about mM after the intracellular Li^+^ concentration reached a steady state. Therefore, once inside the cell, Li^+^ is able to displace Mg^2+^ from its binding sites.


### $Li^+$ INFLUX AND BINDING, AND $Li^+/Mg^{2+}$ COMPETITION IN BOVINE CHROMAFFIN CELL SUSPENSIONS AS STUDIED BY $^7Li$ NMR AND FLUORESCENCE SPECTROSCOPY

Carla P. Fonseca[1,2], Liliana P. Montezinho[1,2], Graça Baltazar[2], Brian Layden[3], Duarte M. Freitas[3], Carlos F. G. C. Geraldes[1,2] and M. Margarida C.A. Castro[1,2*]

[1]Department of Biochemistry and [2]Center for Neuroscience, University of Coimbra, P.O. Box 3126, 3000 Coimbra, Portugal
[3]Chemistry Department, Loyola University of Chicago, Chicago IL 60626, USA

### ABSTRACT

$Li^+$ influx by bovine chromaffin cells, obtained from bovine adrenal medulla, was studied in intact cell suspensions using $^7Li$ NMR spectroscopy with the shift reagent $[Tm(HDOTP)]^{4-}$. The influx rate constants, $k_i$, were determined in the absence and in the presence of two $Na^+$ membrane transport inhibitors. The values obtained indicate that both voltage sensitive $Na^+$ channels and $(Na^+/K^+)$-ATPase play an important role in $Li^+$ uptake by these cells. $^7Li$ NMR $T_1$ and $T_2$ relaxation times for intracellular $Li^+$ in bovine chromaffin cells provided a $T_1/T_2$ ratio of 305, showing that $Li^+$ is highly immobilized due to strong binding to intracellular structures. Using fluorescence spectroscopy and the $Mg^{2+}$ fluorescent probe, furaptra, the free intracellular $Mg^{2+}$ concentration in the bovine chromaffin cells incubated with 15mM LiCl was found to increase by about 1mM after the intracellular $Li^+$ concentration reached a steady state. Therefore, once inside the cell, $Li^+$ is able to displace $Mg^{2+}$ from its binding sites.

### INTRODUCTION

The molecular and cellular mechanisms underlying the clinical use of lithium salts in the treatment of manic-depression (also called bipolar disease) as well as other psychiatric and non-psychiatric conditions are still poorly understood [1-3]. Several interrelated hypotheses have been formulated to clarify the action of $Li^+$. Among these are: 1) $Li^+$ inhibition of the enzymes inositol-1-monophosphatase and adenylate cyclase, causing depletion in brain inositol and cyclic AMP (cAMP) levels [4-7]; and 2) competition between $Li^+$ and $Mg^{2+}$ ions for $Mg^{2+}$ binding sites in biomolecules, in particular guanine-nucleotide binding proteins which are involved in the signal transduction cascade [8-9]. It is possible that the pharmacological action of $Li^+$ cannot be explained by one single mode of action. In an attempt to contribute towards a better understanding of this problem, at the cellular and molecular levels, recent studies using fluorescence spectroscopy with the $Mg^{2+}$ indicator furaptra [10] as well as $^7Li$ and $^{31}P$ NMR spectroscopy [11] have been undertaken. These techniques proved to be useful to investigate $Li^+$ transport [12], $Li^+$ binding [13] and $Li^+/Mg^{2+}$ competition [11,14]. $Li^+/Mg^{2+}$ competition was demonstrated for the phosphate groups of small phosphorylated molecules involved in second messenger systems, such as ATP/ADP, GTP/GDP and $IP_3$ [11,15, 16]; for the phosphate groups of erythrocyte membrane phospholipids [17]; in $Li^+$-loaded human erythrocytes [10,18] and in human SH-SY5Y neuroblastoma cells [14].

Chromaffin cells from the adrenal gland medulla are excitable endocrine cells which can be used as good neuronal models [19,20] and whose neurotransmitter release has been found to be stimulated by $Li^+$ loading [21-23]. Therefore, in this study we used $^7Li$ NMR and fluorescence spectroscopic methods to examine $Li^+$ uptake, intracellular $Li^+$ binding and competition between $Li^+$ and $Mg^{2+}$ ions in bovine chromaffin cells in suspension. The results obtained were compared with those previously obtained with other cell types. These studies aim to establish the generality of the ionic competition model described and may also contribute to the understanding of the pharmacological action of $Li^+$ at the molecular level.

### MATERIALS AND METHODS

### Materials

Collagenase (type B) was purchased from Boehringer Mannheim (Mannheim, Germany). Furaptra and fura-2 (salt and ester forms) and Pluronic® F-127 were obtained from Molecular Probes (Eugene, OR). Percoll was supplied by Pharmacia Biotech AB (Uppsala, Sweden), fetal calf serum by Seromed Biochrom (Berlin, Germany) and the shift reagent $Na_3H_2Tm(DOTP).3NaCl$ by Macrocyclics (Richardson, Texas, USA). All other biochemicals were purchased from Sigma Chemical Company (St.Louis, MO) and inorganic salts and glucose from Merck (Darmstadt, Germany).

### Isolation of bovine chromaffin cells

Chromaffin cells were isolated from bovine adrenal medulla as described by Brocklehurst and Pollard [24] and were purified by centrifugation in a continuous Percoll gradient. The Neutral Red dye test [25] indicated that 65-80% of the cell preparation contained chromaffin cells. Cell viability was tested by the

Trypan Blue dye exclusion method [26]. The cells were maintained in a 1:1 mixture of Dulbecco's Modifed Eagle's Medium / Ham's Nutrient Mixture F-12 (DMEM/F-12) (1.56%) containing 5% of heat-inactivated fetal calf serum, penicillin (10,000 units/ml), streptomycin (100 mg/ml), amphotericin B (25 μg/ml) and 10 μM of cytosine β-D-arabinofuranoside, at 37°C, in a humidified $CO_2$ (5%) and air (95%) atmosphere. The cells were cultured up to a density of $10^6$ cells /ml in 100 mm Petri dishes, 2-4 days before use for NMR and fluorescence experiments. For the fluorescence experiments, the cell preparation was further purified by differential platting in order to reduce contamination by endothelial cells.

**NMR experiments**

To study $Li^+$ influx in bovine chromaffin cells, $10^8$ cells were suspended in a modified Krebs medium (in mM: NaCl 125, LiCl 15, KCl 5, $CaCl_2$ 2, $MgCl_2$ 1, glucose 10, HEPES 20, pH=7.35) containing 7mM of the shift reagent $[Tm(HDOTP)]^{4-}$ (total volume of 1.5ml) and placed in a 10 mm NMR tube (control experiment). To determine if voltage-sensitive $Na^+$ channels and $(Na^+,K^+)$-ATPase contribute to the uptake of $Li^+$, two inhibitors were studied. A specific blocker of the voltage-sensitive $Na^+$ channels, tetrodotoxin (TTX) (1 μM) was used with a preincubation time of 3-4 minutes. To block $(Na^+,K^+)$-ATPase, ouabain (50 μM) was used with a preincubation time of 4-5 minutes.

The NMR experiments were performed with a Varian-Unity 500 NMR spectrometer equipped with a multinuclear 10 mm broad-band probe and a controlled temperature unit. $^7Li$ NMR spectra were acquired at 194.3 MHz at 25°C ± 0.5°C. The samples were spun at 16 Hz to prevent cell sedimentation. $^7Li$ NMR spectra were obtained from 64 transients (total accumulation time of 11minutes), a spectral width of 5500 Hz, a pulse width of 15 μs, an interpulse delay of 10 s and an acquisition time of 0.362 s. The signal-to-noise ratio was enhanced by exponential multiplication with a line broadening of 30 Hz.

$^7Li$ $T_1$ and $T_2$ relaxation measurements for intracellular $Li^+$ were conducted by use of inversion-recovery and Carl-Purcell-Meilboom-Gill (CPMG) pulse sequences, respectively, after the intracellular $Li^+$ concentration reached a steady state at the end of the influx experiments.

The kinetics of $Li^+$ influx is defined by the equation

$$A_\infty - A_t = A_\infty e^{-k_i t} \qquad (1)$$

where $A_t$ and $A_\infty$ are, respectively, the integrals of the intracellular $Li^+$ signal at time (t) and when the $Li^+$ concentration inside the cells reaches the steady state. Thus, the apparent influx rate constants, $k_i$, were determined using a graphical representation of time dependence of the percent of intracellular $Li^+$ from plots of areas of intracellular $Li^+$ signal ($A_i$) over the total area ($A_i + A_e$) ($A_e$ is the area of extracellular $Li^+$ signal), $[A_i/(A_i + A_e)]$ vs. time [12]. The integrals of the NMR signals were calculated using a signal deconvolution program.

**Calculation of total intracellular $[Li^+]_i$ from $^7Li$ NMR spectroscopy**

$^7Li$ NMR spectroscopy was used to determine the total intracellular $Li^+$ concentration

$$[Li^+]_i = ([Li^+]_T (1-C_T) A_i) / (C_T (A_i + A_e)) \qquad (2)$$

where $[Li^+]_i$ and $[Li^+]_T$ are the total intracellular lithium concentrations within the cell and added to the sample, respectively, and $A_i$ and $A_e$ are the areas of the intracellular and extracellular NMR resonances taken at various times. $C_T$ is the cytocrit, i.e., the total percentage volume of cells in the sample used. The volume of cells can be calculated from a cell count and the known volume of a chromaffin cell. In this study, the sample volume was 1.5 ml with a cell concentration of $10^8$. Since these cells are spherical in shape [27] and the diameter is 21.7 μm [27], the volume can be estimated to be ~5400 $μm^3$.

**Fluorescence Spectroscopy experiments**

The fluorescence experiments were performed on a SPEX FluoroMax fluorimeter, at 30°C, using the $Mg^{2+}$ fluorescent dye furaptra.

The free fluorescent probe furaptra has an excitation $\lambda_{max}$ at 370 nm and, as free $Mg^{2+}$ concentration increases, an induced shift to lower frequencies is observed. The excitation $\lambda_{max}$ for the furaptra-$Mg^{2+}$ complex is at 330 nm. The fluorescence intensities were measured at 335 nm and 370 nm and were monitored simultaneously over time with the emission wavelength fixed at 500 nm.

Similar studies were carried out using chromaffin cells permeabilized with digitonin (final concentration 0.2 mM) at 30°C, in a Krebs medium without $Ca^{2+}$ and $Mg^{2+}$ (in mM: NaCl 140, KCl 5, glucose 10, HEPES 20, EGTA 0.5, pH=7.35); increasing concentrations of $Mg^{2+}$ and $Ca^{2+}$ were added to investigate the behavior of furaptra in these conditions.

For the experiments with intact chromaffin cells, 3-4x$10^7$ cells were incubated in a Krebs medium (in mM: NaCl 140, KCl 5, $CaCl_2$ 2, $MgCl_2$ 1, glucose 10, HEPES 20, pH 7.35) containing 1% BSA and 5 μM of the probe in the acetoxymethylester form (furaptra/AM) at 37°C, in a humidified $CO_2$ (5%) / air (95%) atmosphere over 30-40 minutes to allow probe loading. Before addition to the cells, Pluronic® F-127 (0.1%) was added to the previous medium and sonicated over 3 minutes. This detergent was used to help dispersion of the fluorescent indicator in the loading medium [28]. After probe loading, the cells were diluted with Krebs medium with 1% BSA and incubated at 37°C for 30 minutes to allow ester hydrolysis. The cells were then washed in a Krebs medium with 0.1% BSA and finally ressuspended in Krebs medium without BSA.

The intracellular free $Mg^{2+}$ concentrations, $[Mg^{2+}]_i$, were determined through the equation [29]:

$$[Mg^{2+}]_i = \beta K_d (R-R_{min})/(R_{max}-R) \qquad (3)$$

where $K_d$ is the dissociation constant of the furaptra/$Mg^{2+}$ complex (1.5 mM at 37 °C [29]; 1.9 mM at 25°C [14]), R, $R_{min}$ and $R_{max}$ are the ratios of fluorescence intensities at the two wavelengths (335nm/370nm) for the experimental sample, for uncomplexed furaptra (absence of ions) and for furaptra saturated with $Mg^{2+}$, respectively. β is the ratio ($F_{min}/F_{max}$) of the fluorescence intensity at 370 nm for furaptra free of ions ($F_{min}$) and for furaptra saturated with $Mg^{2+}$ ($F_{max}$). The $R_{min}$ and $F_{min}$ parameters were determined in a $Mg^{2+}$-free and $Ca^{2+}$-free solution (in mM: KCl 120, NaCl 20, HEPES 10, EGTA 1, pH 7.35) containing 2 μM of furaptra (salt form). The $R_{max}$ and $F_{max}$ parameters were determined by adding the salt form of furaptra (2 μM) to a $Mg^{2+}$-saturated solution (in mM: $MgCl_2$ 70, KCl 15, NaCl 20, HEPES 10, pH 7.35).

### RESULTS AND DISCUSSION

### $Li^+$ transport across the chromaffin cell membrane

The influx of $Li^+$ into chromaffin cells was studied using $^7Li$ NMR spectroscopy. The paramagnetic shift reagent $[Tm(HDOTP)]^{4-}$ [12] was used to separate $^7Li$ NMR signals corresponding to $Li^+$ inside and outside the cells. Due to its negative charge, this compound is impermeable to cell membranes and shifts the resonance of the extracelullar $Li^+$ to higher frequencies relative to the resonance of the intracellular $Li^+$. **Fig. 1A** shows $^7Li$ NMR spectra from an influx experiment with $10^8$ cells, suspended in a modified Krebs medium containing 15mM LiCl and 7mM $[Tm(HDOTP)]^{4-}$. The signal corresponding to the intracellular $Li^+$ increases with time and reaches a steady state after 60-80 minutes. A graphical representation of the increase in the percent of intracellular $^7Li$ resonance area, $A_i$, relative to the total area of intra and extracellular $^7Li$ resonances, $A_i + A_e$ ($100 \times A_i/(A_i + A_e)$) over time is shown in **Fig. 1B**.

**A)**

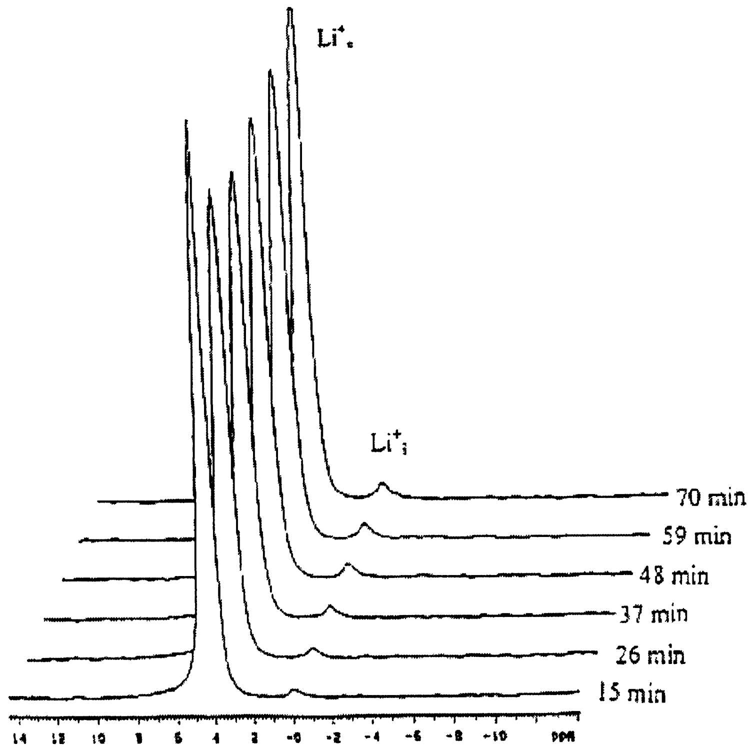

**B)**

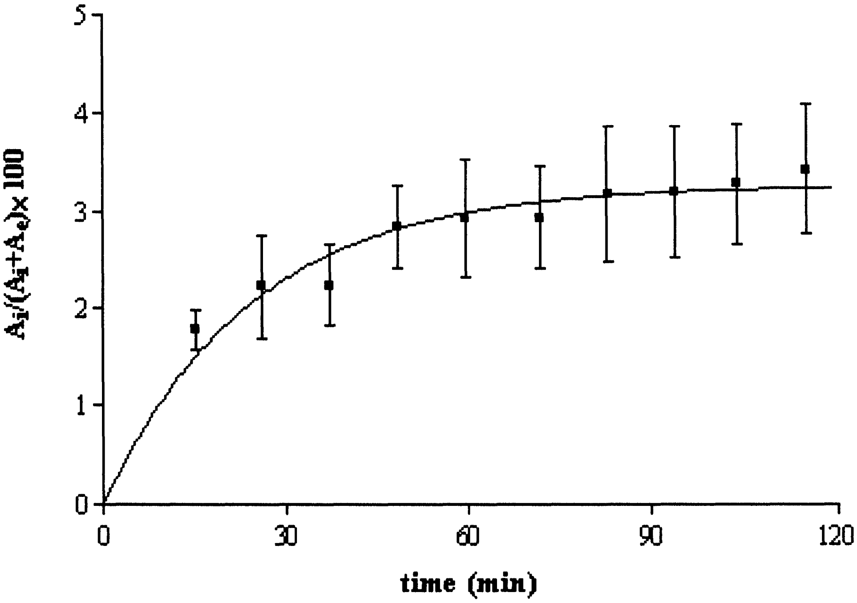

**Figure 1. A)** $^7Li$ NMR spectra are represented over time in an influx experiment with $10^8$ chromaffin cells suspended in a modified Krebs medium containing 15 mM LiCl and 7 mM $[Tm(HDOTP)]^{4-}$. The start of the experiment (t = 0 min) indicates the time when the $Li^+$-free cells were mixed with the $Li^+$-incubation medium. Each spectrum represents the average of the total accumulation time of 11 minutes; **B)** Graphical representation of the time dependence of the percent of intracellular $^7Li^+$ ((100 × $A_i/(A_i + A_e)$) vs. time) in the control experiment.

Similar $^7Li$ NMR experiments were performed in the presence of 1 µM of tetrodotoxin (TTX) and 50 µM of ouabain. **Table I** shows the $k_i$ values determined for the control experiments and for the studies with the two inhibitors.

**Table I**: $Li^+$ influx rate constants, $k_i$, at 25°C, in the absence (control) and in the presence of inhibitors, and respective percent (%) inhibition relative to the control situation. The errors are the standard deviations (SD) of the mean.

| Experimental condition | $k_i$ ($min^{-1}$) (± SD) | % inhibition (relative to the control) |
|---|---|---|
| Control | 0.040 ± 0.003 (n=4) | - |
| TTX | 0.027 ± 0.009 (n=2) | 33 |
| Ouabain | 0.027 ± 0.003 (n=3) | 33 |

The results with TTX indicate that voltage-sensitive $Na^+$ channels are involved in the uptake of $Li^+$ by these cells, confirming the results already obtained with other types of cells [12,30,31]. In the presence of ouabain, an inhibitor of ($Na^+$,$K^+$)-ATPase, an inhibition of 33% in $Li^+$ influx was observed. In human erythrocytes, $Li^+$ is not transported by ($Na^+$,$K^+$)-ATPase under physiological conditions [30]; in these cells $Li^+$ influx is only detected through this pump in the absence of extracellular $Na^+$. However, $Li^+$ influx is detected through ($Na^+$,$K^+$)-ATPase under physiological conditions in some other cell types such as neuroblastoma x glioma hybrid cells [32]. Therefore, the results obtained in this work could be due to a direct or an indirect effect on ($Na^+$,$K^+$)-ATPase. An indirect effect of this inhibitor could occur by changing the $Na^+$ equilibrium or the membrane potential, and therefore indirectly causing a change in $Li^+$ influx. Thus, it should be further investigated if ($Na^+$,$K^+$)-ATPase has a direct or an indirect role in the $Li^+$ transport in this cell type.

To qualitatively compare the amount of $Li^+$ accumulated by this cell type as compared to other cells, we used previously reported studies on human erythrocytes and human neuroblastoma cells. For a meaningful comparison, the cytocrit, the extracellular $Li^+$ concentration and the periods of incubation should be approximately equal. In this study, bovine chromaffin cells at 25 % cytocrit after 80 min accumulate an intracellular $Li^+$ concentration of 1.67 ± 0.43 mM with an extracellular $Li^+$ concentration of 15 mM LiCl as obtained by equation (2). With human neuroblastoma cells (10-15 % cytocrit), an intracellular $Li^+$ concentration of 3.1 ± 0.1 mM was obtained with an extracellular $Li^+$ concentration of 15 mM at 90 min [33]. Unfortunately, no studies with an extracellular $Li^+$ concentration of 15 mM and a similar hematocrit have been performed on human erythrocytes. However, in one study, an extracellular $Li^+$ concentration of 150 mM resulted in an intracellular $Li^+$ concentration of 3 mM after 75 min (10% hematocrit) [13]. Even though the experimental conditions were different in our study as compared to the other two [13,33], it is apparent that human erythrocytes accumulate the least amount of $Li^+$; human neuroblastoma cells and bovine chromaffin cells are more similar in the amount of $Li^+$ accumulated.

**Intracellular $Li^+$ binding**

At the end of the $Li^+$ influx experiments, when the $Li^+$ concentration inside the cell reached the equilibrium, $^7$Li NMR $T_1$ and $T_2$ relaxation times for the $Li^+_i$ resonance were determined using the inversion-recovery and CPMG pulse sequences, respectively. Because $^7$Li NMR $T_1/T_2$ ratio is a measure of the relative degree of immobilization of the $Li^+$ ion, the higher the value of this ratio is, the lower the mobility of $Li^+$ [13]. Therefore, $^7$Li NMR relaxation measurements give information about the physical state of the ion inside the cell. A comparison of the $^7$Li NMR $T_1/T_2$ ratios obtained in this work with those for other types of cells and some of their components is shown in **Table II**. From these values which were obtained with similar intracellular $Li^+$ concentrations [12,13,33], it is possible to conclude that $Li^+$ is more immobilized within chromaffin cells that in other cellular systems already studied [12,13], which is in agreement with the higher degree of complexity of these cells. $Li^+$ is strongly bound to the membrane, intracellular structures in the cytosol and probably inside the chromaffin granules. The high $^7$Li NMR $T_1/T_2$ ratio of the plasma membrane of the human neuroblastoma cells and human erythrocytes as compared to LiCl free in solution (**Table II**) indicates that these are major $Li^+$ binding sites. A more detailed study with various chromaffin cell components, in particular plasma membrane suspensions, could be useful to better define which are the most probable $Li^+$ binding sites in these cells.

**Table II**: $^7$Li NMR $T_1$, $T_2$ and $T_1/T_2$ values for intracellular $Li^+$ in chromaffin cells, compared with other types of cells and their components.

| Sample | $T_1$ (s) (± SD) | $T_2$(s) (± SD) | $T_1/T_2$ |
|---|---|---|---|
| Bovine chromaffin cells | 6.1 ± 0.2 (n=5) | 0.02 ± 0.002 (n=5) | 305 |
| Neuroblastoma Cells [12] | 5.1 ± 0.8 (n=4) | 0.05 ± 0.020 (n=4) | 100 |
| Human erythrocytes [13] | 6.5 ± 0.2 (n=4) | 0.46 ± 0.010 (n=4) | 14 |
| Neuroblastoma Plasma Membranes [12] | 7.2 ± 0.3 (n=4) | 0.17 ± 0.010 (n=4) | 42 |
| Erythrocytes Plasma Membranes [13] | 4.2 ± 0.1 (n=4) | 0.08 ± 0.010 (n=4) | 52 |
| LiCl aq. (viscosity adjusted) [13] | 3.9 ± 0.4 | 3.6 ± 0.6 | 1.1 |

**$Li^+/Mg^{2+}$ competition within chromaffin cells**

Competition between $Li^+$ and $Mg^{2+}$ for $Mg^{2+}$ binding sites in chromaffin cells was studied by fluorescence spectroscopy using the fluorescent probe furaptra. To examine if $Li^+/Mg^{2+}$ competition occurs in $Li^+$-loaded chromaffin cells, $Li^+$ influx experiments were carried out with $2 \times 10^6$ cells, previously loaded with the acetoxymethylester form of furaptra (furaptra/AM), and suspended in a modified Krebs medium containing 15 mM LiCl. The fluorescence excitation spectra in **Fig 2A**, obtained over time during $Li^+$ influx, show an increase in the fluorescence intensity ratio R(335/370) which is related to an increase in the intracellular free $Mg^{2+}$ concentration during $Li^+$ loading. Due to some release of the fluorescent indicator from the cells with time, and its binding to divalent cations present in the extracellular medium, a control experiment was made in the same conditions but with the cells suspended in a Krebs medium without $Li^+$ (data not shown). The fluorescence changes observed during this control experiment were taken into account and subtracted to the $Li^+$-loaded cells data. The fluorescence data was converted into intracellular free $Mg^{2+}$ concentrations through equation (3), using a value of 1.9 mM for $K_d$ (furaptra-$Mg^{2+}$) [14], and the result is represented in **Fig. 2B**.

A)

B)

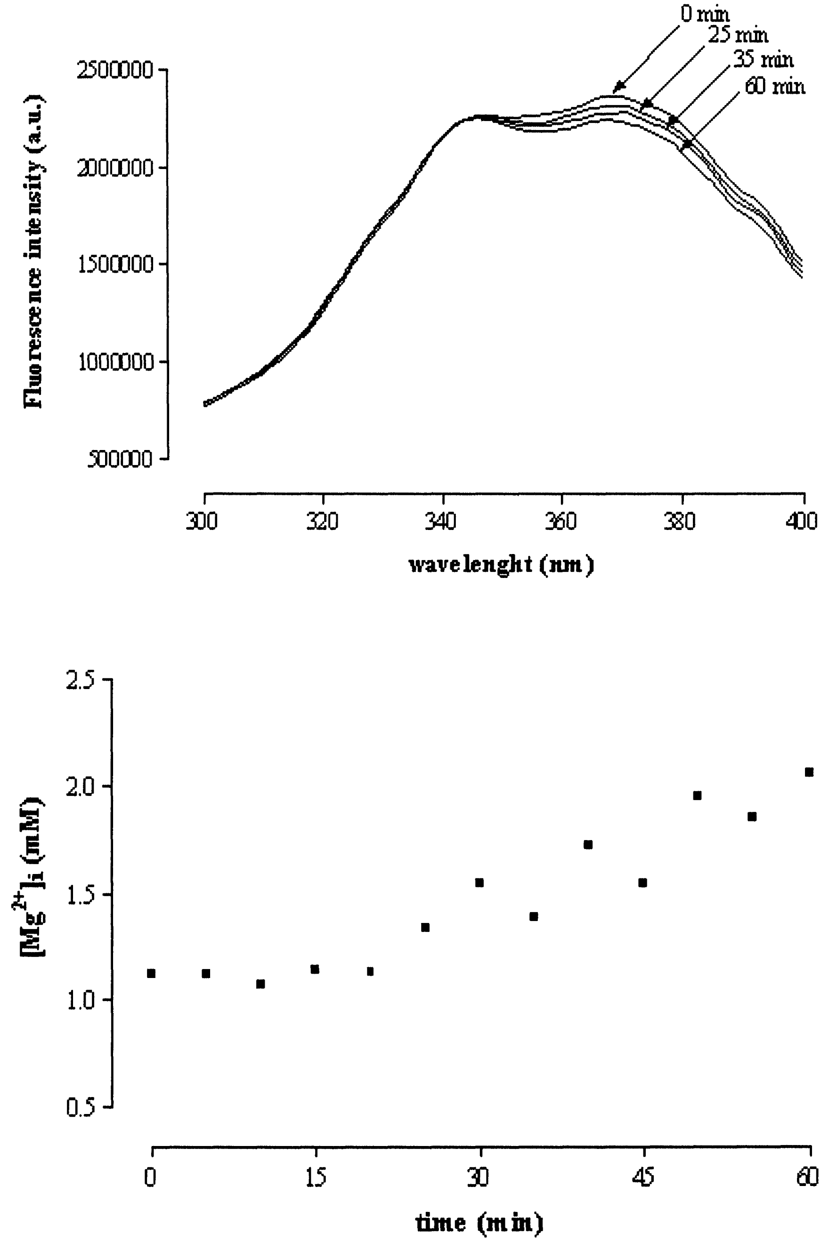

**Figure 2. A)** Fluorescence excitation spectra obtained at 30 °C with 2 × $10^6$ chromaffin cells preincubated with furaptra/AM and suspended in a modified Krebs medium containing 15 mM LiCl (in mM: NaCl 125, LiCl 15, KCl 5, $CaCl_2$ 2, $MgCl_2$ 1, glucose 10, HEPES 20, pH 7.35). The emission wavelength was fixed at 500 nm; **B)** Graphical representation of the time dependence of the intracellular free $Mg^{2+}$ concentration in chromaffin cells, during the influx experiment described in A). The intracellular free $Mg^{2+}$ concentrations were calculated using eq. 3 and a value of 1.9 mM for $K_d$ (furaptra-$Mg^{2+}$) [14].

As shown in **Fig. 2B**, there is an increase in the intracellular free $Mg^{2+}$ concentration with time, from its basal value of 1.1 ± 0.20 mM (n=3), determined in the absence of $Li^+$, to 2.1 ± 0.14 mM (n=2), after the incubation of the cells in a 15 mM LiCl modified Krebs medium for 60 minutes.

As the intracellular space of the $Li^+$-loaded cells contains $Mg^{2+}$, $Ca^{2+}$ and $Li^+$, their binding to the dye has to be taken into account. *In vitro* studies of the excitation fluorescence spectra of furaptra, in its salt form, in the presence of different concentrations of $Mg^{2+}$ (in the absence of $Ca^{2+}$), and of different concentrations of $Ca^{2+}$ (in the absence of $Mg^{2+}$) have been described in the literature [29,34]. The behavior of the probe in the presence of $Ca^{2+}$ or $Mg^{2+}$ is very similar, with an excitation $\lambda_{max}$ for the furaptra-$Ca^{2+}$ complex also at 330 nm and the dissociation constants $K_d$ for furaptra-$Ca^{2+}$ and furaptra-$Mg^{2+}$ complexes are 53 μM [29,34] and 1.5 mM [29] (or 1.9 mM [14]), respectively. Fluorescence excitation spectra obtained with $2 \times 10^6$ chromaffin cells previously incubated with furaptra/AM, suspended in a free-$Ca^{2+}$ and free-$Mg^{2+}$ Krebs medium and then permeabilized with digitonin, were found to be very similar to those obtained in solution, when increasing amounts of $Ca^{2+}$ and $Mg^{2+}$ were added (data not shown). Thus, the fluorescent probe has the same photophysical properties inside the cells and free in solution.

$Ca^{2+}$ has a higher affinity to furaptra than $Mg^{2+}$ according to the respective $K_d$ values [29,34]. However, it was observed that the intracellular free $Ca^{2+}$ levels during $Li^+$-loading experiments with chromaffin cells using the $Ca^{2+}$ sensitive fluorescent probe fura-2, increase slowly from its basal value (100-200 nM) up to around 600 nM (data not shown). Once the induced shift of furaptra to lower frequencies occurs when $Ca^{2+}$ concentration is higher than 1μM [29], this ion do not interfere with the spectroscopic properties of this probe.

Since $Li^+$ has also been shown to bind furaptra ($K_d$ = 250 mM at 25 °C and 237 mM at 37 °C [10]) it was necessary to determine if the contribution of $Li^+$ binding to the dye was significant and if a correction in eq. 3 was necessary [11]. In this study it was already mentioned that the intracellular $Li^+$ concentration was estimated to be 1.67 ± 0.43 mM. This amount of $Li^+$ would only affect furaptra fluorescent properties by 0.7 % (1.67 mM/250 mM), which can not account for the changes shown in **Fig. 2B**.

These data show that the increase in the ratio of fluorescence intensities R(335/370) of furaptra observed when $Li^+$ goes into the cells is due to an increase in the intracellular free $Mg^{2+}$ concentration, without interference of cytosolic $Ca^{2+}$ or $Li^+$ in the fluorescent properties of this probe, confirming the ability of $Li^+$ to displace $Mg^{2+}$ from its binding sites within the cells.

### CONCLUSIONS

The present study investigates $Li^+$ influx pathways in bovine chromaffin cell suspensions by $^7$Li NMR methods. The data indicate that voltage-sensitive $Na^+$ channels, which have been shown to be active in $Li^+$ influx in human neuroblastoma SH-SY5Y cells [12] and other cells [30,31], play an important role in $Li^+$ uptake by this cellular system. Also, ($Na^+$,$K^+$)-ATPase affects $Li^+$ influx rates when inhibited by ouabain. However, the exact role of this pathway for $Li^+$ should be further explored with this cell type. Comparing the total accumulation of $Li^+$ under similar conditions, it is apparent that human erythrocytes accumulate much less $Li^+$ than human neuroblastoma and chromaffin cells. $^7$Li NMR $T_1/T_2$ ratios for intracellular $Li^+$ in chromaffin cells show that $Li^+$ is highly immobilized due to strong binding to intracellular structures as compared to other cell types. A more detailed study of the $Li^+$ binding sites will help elucidate the location of major $Li^+$ binding sites in bovine chromaffin cells. Once inside the cell, $Li^+$ is able to displace $Mg^{2+}$ from its binding sites. Thus, in this work, we confirm that $Li^+/Mg^{2+}$ competition also occurs in bovine chromaffin cells, in agreement with previous results published in the literature with other cellular systems [14,18], which is one of the hypotheses to explain the therapeutic action of $Li^+$ at the molecular and cellular levels.

### ACKNOWLEDGEMENTS

M.M.C.A.Castro and C.F.G.C.Geraldes acknowledge financial support from F.C.T, Portugal (grant PECS/P/SAU166/95) and D.M.Freitas and B.Layden from National Institute of Mental Health, USA (grant MH-45926). C.P.Fonseca and L.P.Montezinho were supported by F.C.T. grants Praxis XXI/BD/21462/99 and SFRH/BD/3286/2000, respectively.

### REFERENCES

1. N.J. Birch, in *Metal Ions in Biological Systems*. H.Sigel (ed.) (1982) Vol.14, 257-313, Marcel Dekker, Inc., New York.
2. R.H. Lenox, R.K. McNamara, R.L. Papke, H.K. Manji, *J.Clin. Psychiatry* (1998) **59**, 37- 47.
3. R.S. Jope, *Mol. Psychiatry* (1999) **4**, 117-128.
4. J.H. Allison, M.A. Stewart, *Nature* (1971) **233**, 267-268.
5. L.M. Hallcher, W.R. Sherman, *J. Biol. Chem.* (1980) **255**,10896-10901.
6. M.J. Berridge, C.P. Downes, M.R. Hanley, *Cell* (1989) **59**, 411-419.
7. V.Saudek, P.Vincendon, Q.T. Do, A.Atkinson,V. Sklenár, P.D. Pelton, F. Piriou, A.J. Ganzhorn, *Eur. J. Biochem.* (1996) **240**, 288-291.
8. S. Avissar, G. Schreiber, A. Danon, R.H. Belmaker, *Nature* (1988) **331**, 440-442.
9. S. Avissar, G. Schrieber, *Biol. Psychiatry* (1992) **31**, 435-459.
10. D.M. Freitas, L. Amari, C. Srinivasan, Q. Rong, R. Ramasamy, A. Abraha, C.F.G.C. Geraldes, M.K. Boyd, *Biochemistry* (1994) **33**, 4101- 4110.
11. L.Amari, B. Layden, Q. Rong, C.F.G.C. Geraldes, D.M. Freitas, *Anal. Biochem.* (1999) **272**, 1-7.
12. J. Nikolakopoulos, C. Zachariah, D.M. Freitas, E.B.Stubbs, Jr., R. Ramasamy, M.M.C.A. Castro, C.F.G.C. Geraldes, *J. Neurochem.* (1998) **71**,1676-1684.
13. Q. Rong, M. Espanol, D.M. Freitas, C.F.G.C.Geraldes, *Biochemistry* (1993) **32**, 13490-13498.

14. L. Amari, B. Layden, J. Nikolakopoulos, Q. Rong, D.M. Freitas, G. Baltazar, M.M.C.A. Castro, C.F.G.C. Geraldes, *Biophys. J.*, (1999) **76**, 2934-2942.
15. Q.Rong, D.M. Freitas, C.F.G.C. Geraldes, *Lithium* (1992) **3**, 213-220.
16. Q. Rong, D.M. Freitas,C.F.G.C. Geraldes, *Lithium* (1994) **5**, 147-156.
17. C. Srinivasan, N. Minadeo, C.F.G.C. Geraldes, D.M. Freitas, *Lipids* (1999) **34**,1211-1221.
18. R. Ramasamy, D.M. Freitas, *FEBS Lett.* (1989) **244**, 223-226.
19. S. W. Carmichael, *Trends Pharmacol. Sci.* (1982) **3**, 308-310.
20. J. M. Trifaró, *Trends Pharmacol. Sci.* (1982) **3**, 389-392.
21. F.J. Abajo, M.A. Serrano-Castro, B. Garijo, P. Sánchez-Garcia, *Br. J. Pharmac.* (1987) **91**, 539-546.
22. F.J. Abajo, M.A. Serrano-Castro, P. Sánchez-Garcia, *Ann. N. Y. Acad. Sci.* (1961) **639**, 665-667.
23. M.T. de la Fuente, R. Maroto, E. Esquerro, P. Sanchez-Garcia, A.G. Garcia, *Eur. J. Pharmacol.* (1996) **306**, 219-226.
24. K.W. Brocklehurst, H.B. Pollard, in *Peptide Hormones: a Practical Approach,* K. Siddle and J. Hutton (eds.) (1990) 233-255, IRL Press, Oxford.
25. L.W.Role and R.L.Perlman, *J. Neurosci. Methods* (1980) **2**, 253-265.
26. M.K.Jr. Patterson, *Methods Enzymol.* (1979) **58**, 141-152.
27. G.Q. Fox, *Cell Tissue Res.* (1996) **284**, 303-316.
28. L.B. Cohen, B.M. Salzberg, H.V. Davila, W.N. Ross, D.Landowne, A.S. Waggoner, C.H. Wang, *J. Memb. Biol.* (1974) **19**, 1-36.
29. B. Raju, E. Murphy, L.A. Levy, R.D. Hall and R.E. London, *Am. J. Physiol.* (1989) **256**, C540-C548.
30. B.E. Ehrlich, J.M. Diamond, *J. Memb. Biol.* (1980) **52,** 187-200.
31. M. Kato, P.-M. Lledo, J.-D. Vincent, *Am. J. Physiol.* (1991) **261**, C218-C223.
32. G. Reiser, J. Duhm, *Brain Res.* (1982) **252**, 247-258.
33. B. Layden, C. Diven, N. Minadeo, F.B. Bryant, D. Mota de Freitas, *Bipolar Disorders* (2000) **2**, 200-204.
34. R.E. London, *Annu. Rev. Physiol.* (1991) **53,** 241-258.

**Received: February 21, 2001 – Accepted: February 28, 2001 – Received in revised camera-ready format: March 1, 2001**

